# Clinical therapeutic effects of probiotics in patients with constipation associated with Parkinson disease: A protocol for systematic review and meta-analysis

**DOI:** 10.1097/MD.0000000000027705

**Published:** 2021-11-05

**Authors:** Xiaoyun Yang, Runjin Zhou, Wenhui Di, Qian He, Qingwei Huo

**Affiliations:** Medical College of Acupuncture-Moxibustion and Rehabilitation, Guangzhou University of Chinese Medicine, Guangzhou, China.

**Keywords:** constipation, meta-analysis, Parkinson disease, probiotics

## Abstract

**Background:**

Constipation is the most predominant symptom of Parkinson disease (PD), preceding the occurrence of motor symptoms in some patients, leading to reduced quality of life (QOL). The general approaches for the treatment have some side effects, but probiotics are live or attenuated microorganisms attributed to ameliorating constipation effects. Moreover, as treatments are generally well tolerated and side effects are scarce, there is room for further research. Therefore this work aims at investigating the clinical effectiveness and safety of probiotics for constipation in PD.

**Methods:**

Published RCTs will be retrieved by searching Medline, Embase, Cochrane Library, Web of Science, China National Knowledge Infrastructure (CNKI), VIP, Wan Fang database, and China Biology Medicine Database (complete bowel movement), which will be searched from establishment of the database to October 10, 2021. Preferred Reporting Items for Systematic Review and Meta-Analysis Protocols (PRISMA-P) guidelines are used to design this protocol. RevMan V.5.3 software will be used for meta-analysis, risk of bias will be assessed by the Cochrane Collaboration tool and the collected evidence will be narratively synthesized. We will also perform a meta-analysis to pool estimates from studies considered to be homogenous. Subgroup analyses will be based on intervention or overall bias.

**Conclusion:**

The meta-analysis will assess the effectiveness and safety of using probiotics to treat and heal the constipation of PD.

**Ethics and dissemination:**

Ethics approval is unrequired.

**Registration number:**

CRD42021276215.

## 1 Introduction

Parkinson disease (PD) is a neurodegenerative disease, with an overall prevalence of 17% in people over 65 years old,^[1]^ and clinical manifestations of both motor and non-motor symptoms.^[2–4]^ More and more attention has been paid to the study of non-motor symptoms, including concurrent cognitive dysfunction, swallowing dysfunction, constipation, and fatigue.^[5]^ Constipation is the most common one, and the incidence in the population with PD ranges from 27.10% to 70.39%.^[6]^ The decrease of defecation times and the change of stool traits became the main reasons patients sought treatment. Constipation usually occurs before the motor symptoms.^[7,8]^ Most patients visit the gastroenterology department, which makes the diagnosis of the disease difficult. Recognizing the pre-motor factors in PD and determining the mechanisms regulating them may provide the possibility for early diagnosis and more timely therapeutic intervention, thus delaying or even preventing the development of progressive motor symptoms in PD.^[9]^

The composition of the intestinal flora is different in patients with constipation and ordinary people.^[10]^ Although the effect of probiotics on the intestinal flora of patients with constipation is not apparent, a few experiments have proved that the composition of the intestinal flora has changed significantly after the supplementation of probiotics.^[11]^ Recently, studies have reported that intestinal microbiota changes are related to PD.^[12–14]^ Intestinal inflammation and intestinal permeability increase with age. The circulation of intestinal microorganisms aggravates systemic inflammation. The inflammatory response promotes the degradation of intestinal barrier function, forming a vicious circle.^[15]^ The intestinal flora changes will change the levels of hormones, neurotransmitters,^[16,17]^ and signaling molecules in the nervous system. The immunoregulation of intestinal–brain interaction may be related to the pathology of neurodegenerative diseases, such as multiple sclerosis, autism, depression, schizophrenia, and PD.^[18]^ Some studies have shown that normal intestinal flora and the intake of probiotics can change the levels of metabolites related to the metabolism of amino acids and polysaccharides in the blood.^[19,20]^ In contrast, probiotics induce the intestinal flora to produce signaling molecules such as tryptophan metabolites, aminobutyric acid, and other neuroactive substances.^[21,22]^ These molecules regulate the intestinal environment, thus changing the interaction between the intestine and the brain. Therefore, the regulation of intestinal microflora is a potential therapeutic target for PD.^[23]^

This study outlines a systematic review and meta-analysis of the efficacy of probiotics in patients with PD constipation. The potential control group was the placebo group. The primary outcome measures were complete defecation, quality of life, and adverse events. This study will provide an objective clinical basis for the effectiveness and safety of probiotics in treating constipation in PD.

## 2 Objectives

This protocol proposes a systematic review and meta-analysis method to provide clinical evidence of the benefits of probiotics to patients with PD. This protocol may provide evidence and form treatment recommendations for clinicians.

## 3 Methods

### 3.1 Inclusion criteria

#### 3.1.1 Studies

Randomized controlled trials of probiotics in patients with constipation associated with PD will be included.

#### 3.1.2 Participants

Diagnosis of idiopathic PD^[24]^; men and women between the ages of 50 and 80 years; Hoehn and Yahr (H&Y) stage I–III^[25]^; ability to walk unassisted for the required gait tasks; on a stable dose of antiparkinsonian medication for at least 2 weeks before beginning the study; able to follow simple commands and to have no uncontrolled chronic diseases; fulfilled Rome I–IV criteria for functional constipation.

#### 3.1.3 Interventions

##### 3.1.3.1 Experimental interventions

Probiotics or (and) prebiotic.

##### 3.1.3.2 Comparator interventions

Placebo control.

#### 3.1.4 Outcome measures

##### 3.1.4.1 Primary outcomes

The average number of spontaneous bowel movements (SBM) per week, number of adverse events.

##### 3.1.4.2 Secondary outcomes

The improvement in stool consistency, satisfaction with treatment.

### 3.2 Search strategy

#### 3.2.1 Electronic searches

Published RCTs will be retrieved by searching Medline, Embase, Cochrane Library, Web of Science, China National Knowledge Infrastructure (CNKI), VIP, Wan Fang database, China Biology Medicine Database (complete bowel movement). We will use the following search terms: randomized controlled trial, probiotics, prebiotic, Parkinson disease, parkinsonism, paralysis agitans, constipation. See Table 1 for the search strategy.

**Table 1:** Search strategy used in PubMed database.

No.	Search items
1	random?ed
2	randomized controlled trial
3	controlled clinical trial
4	placebo
5	clinical trials
6	randomly
7	trial
8	1 or 2–7
9	Parkinson's disease
10	Idiopathic Parkinson's disease
11	Lewy body Parkinson's disease
12	Parkinson's disease, idiopathic
13	Parkinson's disease, Lewy body
14	Parkinson disease
15	Parkinson disease, idiopathic
16	Idiopathic Parkinson disease
17	Lewy body Parkinson disease
18	Primary Parkinsonism
19	Parkinsonism, primary
20	Paralysis agitans
21	Tremor paralysis
22	Shaking palsy
23	9 or 10–22
24	Constipation
25	Functional constipation
26	Dyschezia
27	Colonic inertia
28	Astriction
29	Obstipation
30	24 or 25–29
31	Prebiotics
32	Prebiotic
33	Probiotics
34	Probiotic
35	31 or 32–34
36	8 and 23 and 30 and 35

### 3.3 Data collection and analysis

#### 3.3.1 Selection of studies

**Figure 1:**
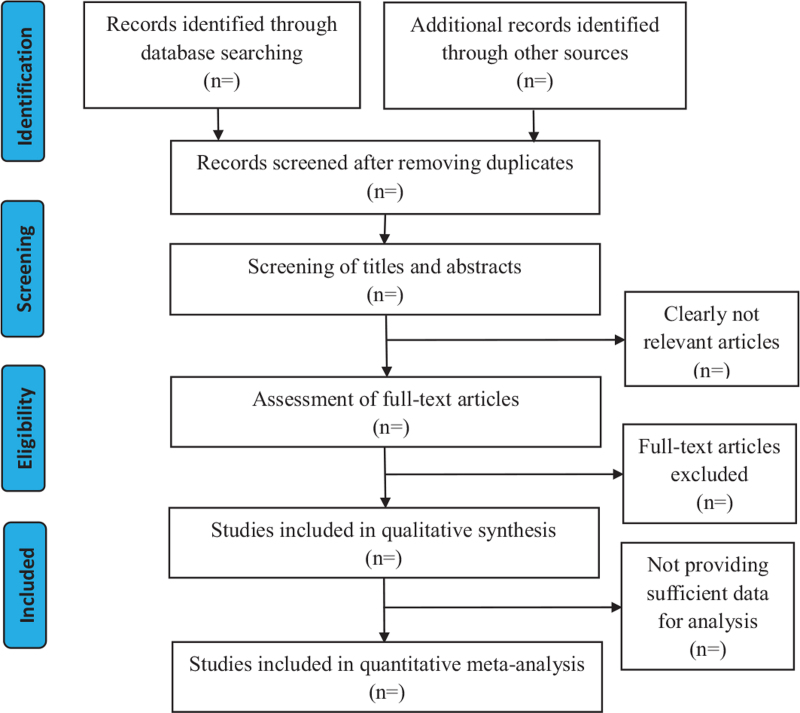
Study flow diagram.

The 2 authors will review and screen the titles and abstracts to identify eligible trials. According to the inclusion criteria, duplicates will be removed with EndNote (v.x9.0). If necessary, the full text will be read, and the exclusion studies will be listed in a table; reasons are given for the exclusion, if there are any differences, will be discussed with a methodology expert. See Fig. 1 for the study flow diagram.

#### 3.3.2 Data extraction and management

Both authors will use the data extraction form to extract the participants. The following information will be extracted according to the CONSORT statement format: general information (author, year of publication, title, abstract), research methods (trial design, subjects, interventions, outcomes, randomization, blinding), results (number of randomized cases, subjects, baseline data, number of included cases and adverse reactions). We will enter the data into Review Manager (RevMan V.5.3).

#### 3.3.3 Assessment of risk of bias in included studies

The risk of bias in 6 areas (sequence generation, allocation hiding, blindness, incomplete data evaluation, selective results reporting, and other sources of bias) will be assessed with the Cochrane Deviation Risk Collaborative Tool.^[26]^ This tool will provide reasons to judge potential risks.

#### 3.3.4 Treatment effect measures

A risk ratio based on a 95% CI will represent the evaluation of the effect of the dichotomous results. We will represent the estimated effect as the average difference within a 95% CI for continuous results. The weighted mean difference will be used for data measured at the same scale and using the same units. Otherwise, a standardized mean difference will be used.

#### 3.3.5 Heterogeneity assessment

We will use *I*^2^ statistics to quantify the inconsistencies between the included studies. The study will not be considered heterogeneous when an *I*^2^ value is <50%; an *I*^2^ value >50% indicates the significant statistical difference between the trials. Qualitative subgroup analysis will be performed to explore possible causes.

#### 3.3.6 Missing data

In cases of insufficient data from an experiment, we will try to contact the first author or the corresponding author of the included study. We will also use library resources to retrieve missing or insufficient data manually. If possible, an intent-to-treat analysis should be conducted, including data for all participants in the group to which they were initially randomly assigned. Additionally, a sensitivity analysis should be conducted to determine whether the results are consistent.

#### 3.3.7 Assessment of reporting biases

Bias and small-scale studies will be detected with funnel plots. If the meta-analysis includes >10 studies, the asymmetry will be explained with the Egger method.^[27]^

#### 3.3.8 Data synthesis

If a meta-analysis is possible, the results of the binary data will be expressed as risk ratios (RR) using RevMan V.5.3, and the results will be expressed as mean differences (MD) for continuous data. If *I*^2^ test results are <50%, the data will be synthesized using a fixed-effects model. If they are between 50% and 75%, the data will be synthesized using a random-effects model. If they are over 75%, we will investigate possible causes from a clinical and methodological perspective and perform subgroup analysis.

#### 3.3.9 Subgroup analysis

Where the data are available, we plan to do meta-regression or subgroups analysis to test the influence of the following factors on the treatment: different treatment durations of probiotics (within 2 weeks, 4 weeks, 6 weeks).

#### 3.3.10 Sensitivity analysis

We will conduct a sensitivity analysis to verify the robustness of the research conclusions, assess the methodological quality, the study design, the effect of sample size and missing data, and the effect of the analysis method on the results of this review. The meta-analysis will be repeated, and lower-quality studies will be excluded. These results will then be compared and discussed.

#### 3.3.11 Ethics and dissemination

This study requires formal ethics approval, as the data to be used is not personal data and does not involve privacy. This article will evaluate the effects of probiotics on PD. Results will be disseminated through peer-reviewed publications or at relevant conferences.

## 4 Discussion

Meta-analysis shows that probiotics can relieve constipation, increase the number of complete defecation, improve the quality of life, and reduce the number of adverse events. When the research amount is enough, we will make a meta-analysis of the UPDRSIII scale to further clarify the improvement of probiotics on non-motor symptoms of PD. At present, the research distribution of probiotics in treating Parkinson constipation has not been concentrated, and there are differences in language and genetic background of different countries, which quickly lead to publication and language deviation. In addition, it is difficult to assess whether a single probiotic has the same therapeutic effect on patients in different areas because of their different constipation, eating habits, drugs, intestinal microflora, and living environment. More importantly, there are many strains of probiotics, and individual strains of different probiotics may have different effects, so more research needs to be collected to evaluate the efficiency of different strains. Later, we will conduct a subgroup analysis to reduce this difference. In view of the current problems, a comprehensive meta-analysis is needed to evaluate the therapeutic effect of probiotics on the constipation of Parkinson patients. This study included all randomized controlled trials of probiotics in the treatment of PD constipation, providing evidence for clinical patients with Parkinson constipation.

## Author contributions

Qingwei Huo is the guarantor of this article and will act as an arbitrator in the event of a dispute. Runjin Zhou and Xiaoyun Yang have established search strategies. Runjin Zhou and Xiaoyun Yang will independently complete the research selection, data extraction, and risk of bias assessment. Wenhui Di and Qingwei Huo will perform data synthesis. Subsequent and final versions of the plan have been rigorously reviewed, modified, and authorized by all authors.

**Conceptualization:** Qingwei Huo.

**Data curation:** Xiaoyun Yang, Runjin Zhou.

**Formal analysis:** Xiaoyun Yang, Runjin Zhou.

**Funding acquisition:** Qingwei Huo.

**Investigation:** Xiaoyun Yang, Runjin Zhou.

**Methodology:** Wenhui Di, Qian He.

**Project administration:** Qingwei Huo.

**Resources:** Xiaoyun Yang.

**Software:** Xiaoyun Yang, Runjin Zhou.

**Visualization:** Wenhui Di, Qian He.

**Writing – original draft:** Xiaoyun Yang, Runjin Zhou.

**Writing – review & editing:** Qingwei Huo.
